# Senior citizens as rescuers: Is reduced knowledge the reason for omitted lay-resuscitation-attempts? Results from a representative survey with 2004 interviews

**DOI:** 10.1371/journal.pone.0178938

**Published:** 2017-06-12

**Authors:** Peter Brinkrolf, Andreas Bohn, Roman-Patrik Lukas, Marko Heyse, Thomas Dierschke, Hugo Karel Van Aken, Klaus Hahnenkamp

**Affiliations:** 1 Department of Anaesthesiology, Greifswald University Hospital, Greifswald, Germany; 2 Emergency Service, City of Münster Fire Service, Münster, Germany; 3 Department of Anaesthesiology, Intensive Care and Pain Therapy, Münster University Hospital, Münster, Germany; 4 Institute of Sociology, University of Münster, Münster, Germany; University of Milano, ITALY

## Abstract

**Objective:**

Resuscitation (CPR) provided by a bystander prior to the arrival of the emergency services is a beneficial factor for surviving a cardiac arrest (CA). Our registry-based data show, that older patients receive bystander-CPR less frequently. Little is known on possible reasons for this finding. We sought to investigate the hypothesis that awareness of CPR measures is lower in older laypersons being a possible reason for less CPR-attempts in senior citizens.

**Methods:**

1206 datasets on bystander resuscitations actually carried out were analyzed for age-dependent differences. Subsequently, we investigated whether the knowledge required carrying out bystander-CPR and the self-confidence to do so differ between younger and older citizens using computer-assisted telephone interviewing. 2004 interviews were performed and statistically analyzed.

**Results:**

A lower level of knowledge to carry out bystander-CPR was seen in older individuals. For example, 82.4% of interviewees under 65 years of age, knew the correct emergency number. In this group, 66.6% named CPR as the relevant procedure in CA. Among older individuals these responses were only given by 75.1% and 49.5% (V = 0.082; P < 0.001 and V = 0.0157; P < 0.001). Additionally, a difference concerning participants’ confidence in their own abilities was detectable. 58.0% of the persons younger than 65 years were confident that they would detect a CA in comparison to 44.6% of the participants older than 65 years (V = 0.120; P < 0.001). Similarly, 62.7% of the interviewees younger than 65 were certain to know what to do during CPR compared to 51.3% of the other group (V = 0.103; P < 0.001).

**Conclusions:**

Lower levels of older bystanders' knowledge and self-confidence might provide an explanation for why older patients receive bystander-CPR less frequently. Further investigation is necessary to identify causal connections and optimum ways to empower bystander resuscitation.

## Introduction

The average global incidence of out-of-hospital cardiac arrest in adults treated by emergency medical services is about 60 per 100.000 person-years [1]. Patients who suffer cardiac arrest have a poor prognosis; in a meta-analysis by Sasson et al., the rate of survival to hospital discharge among 142,740 patients included in the study was 7.6% (95% CI, 6.7 to 8.4%) [2]. The prognosis improved significantly between 2002 and 2011, but despite this increase, the 1-year survival rate is still low at 11.8% [3]. However, recent data suggest that much higher survival rates may be possible if optimal care is provided [4].

The probability of surviving cardiac arrest depends on influencing factors, such as the cause of the cardiac arrest [5], the initial cardiac rhythm [6], and the depth [7] and rate [8] of chest compressions. Greater age of the patients is associated with a lower survival rate [9]. However, large follow-up studies show that the quality of life after successful CPR is comparable to the general population. Smith et al. used the twelve-item short form (SF-12) health survey on 687 patients 12 month after successful resuscitation. The authors demonstrated that senior survivors older than 75 years of age even had a slightly better quality of life when compared to the age- and sex-adjusted general population [10].

Resuscitation measures performed by a bystander preceding the arrival of the professional emergency services results in two to threefold higher survival [2, 11–13]. It is known that the majority of cardiac arrests occur in the home setting [14] and are mostly observed by laypersons, who are usually relatives of the patient [15]. Surprisingly, the probability of a resuscitation being initiated by a relative is lower than in other groups of individuals [16, 17]. The rate of resuscitations performed by a bystander [18, 19] and the likelihood of survival [20,21] are lower in the domestic setting than in other cases of cardiac arrest.

It is unclear why older patients receive less bystander-CPR and why patients’ relatives in a domestic setting start resuscitation measures particularly seldom. One possibility might be that potential first-aiders at patients’ homes are themselves older than in other locations such as workplaces, streets or gymnasiums. The hypothesis for the present study was that awareness of lay-resuscitation is lower in the elderly.

## Materials and methods

We performed a representative phone questionnaire survey. The investigation was carried out in Münster, a German 300.000-inhabitant city. The interviews were performed in the form of computer-aided telephone inquiries (CATIs). A questionnaire was used that includes 66 items. Prior to the survey, the questionnaire was validated by cognitive pre-tests using the so-called “Think Aloud” method. Therefore, the questionnaire was read to five individuals who were asked to explain why they chose a specific answer and to name any misleading questions. Thereby, some unclear formulations where removed before starting the survey. Using the final questionnaire, a total of 2004 individuals were contacted by phone in two survey waves (June 2013 and November 2013).

### Sampling

The sample used was drawn from a database managed by the Society for Sociological Infrastructure Institutes (*Gesellschaft Sozialwissenschaftlicher Infrastruktureinrichtungen*, GeSIS), a registered association in Germany. A framework with a Gabler–Häder design [22] which has become established for most phone-based surveys in Germany was used.

### Reflection of the total population

Comparison of the data with the available figures from the city of Münster’s Office for Urban Development, Urban Planning, and Traffic Planning [23] showed that the sex distribution in the sample reflected the total population well; deviations are within the expected margin of error. The age distribution showed greater deviation from the total population: whereas individuals under the age of 35 represented a smaller proportion of the sample (13.5% of the sample vs. 25.7% of the population), those aged over 60 were over-represented (37.8% vs. 25.5%).

### Sample statistics

A total of 8000 phone numbers were available for the interviews and were fully used. Just over 12,000 dialing operations were attempted in order to achieve the 2000 interviews intended. The complete sample statistics are given in Table 1. The uptake rate of 29.4% was within the usual range of 20–30% for phone surveys [24].

**Table 1:** Sample statistics for the interviews.

	Absolute	Relative
Gross sample 1	16000	100% = 16000
Not used	0	0.0%
Number not available	5728	35.8%
Business number	267	1.7%
Data tone	181	1.1%
	6176	38.6%
Gross sample 2	9824	100% = 9824
Answering machine	1296	13.2%
Constant ringing tone	1290	13.1%
Not target group	101	1.0%
Appointment not kept	166	1.7%
Just hung up	158	1.6%
	3011	30.6%
Gross sample 3	6813	100% = 6813
Interview declined	4797	70.4%
Interrupted	12	0.2%
Interview (direct)	1882	27.6%
Interview (indirect)	122	1.8%
Total interviews	2004	29.4%

From a total of 16,000 phone numbers, 6813 numbers were suitable for interviews and 2004 individuals (29.4%) completed an interview

### Statistical analysis

The usual univariate and bivariate procedures, as well as linear regressions, were used in the statistical analysis. Cramér’s *V* was used as a measure of association between nominal variables, the Kendall tau-b coefficient for ordinal variables, and correlation coefficients for metric/quantitative variables.

The chi-squared test was used to assess the significance level, interpreted as follows:

- Significant results: probability of error is < = 5% (marked with one asterisk: *).
- Very significant results: probability of error is < = 1% (marked with two asterisks: **).
- Highly significant results: probability of error is < = 0.5% (marked with three asterisks: ***).

Statistical processing of the data was carried out using IBM SPSS Statistics, version 22.0 (IBM Corporation, Armonk, New York, USA) and Microsoft Excel 2010 (Microsoft Corporation, Redmond, Washington, USA).

### Resuscitation registry

Data on bystander resuscitations actually carried out in Münster were exported from the database of the German Resuscitation Registry. Since May 2007, this database has included data on all out-of-hospital resuscitations reported by emergency physicians in the city of Münster. 156 patients were excluded from the total of 1362 cases identified, as patients were under the age of 18 or the cardiac arrest occurred after the arrival of the emergengy service. The remaining 1206 cases were included in the analysis.

### Ethical considerations, consent

The study was submitted for assessment to the ethics committee of the Medical Council of Westphalia–Lippe and the Medical Faculty of the University of Münster and was processed under case number 2013-317-f-N. Following initial inspection by the ethics committee, it was determined that no ethical aspects were involved and that an ethics vote was not required. Verbal informed consent regarding data collection, procession and publication was obtained from all interviewees before starting the interview. The given answer was documented digitally and the call was either ended or it was preceded with the questionnaire. If the participant provided consent but did not have time to answer the questionnaire right away, a recall at a later date was timed.

## Results

A total of 2004 interviews were conducted. The interview participants’ sociodemographic data are shown in Table 2. The participants’ average age was just under 57, with a range of 18–91 years.

**Table 2:** Socio-economic data for the participants.

	Absolute	Relative
*Sex*		
Female	1106	55.2%
Male	898	44.8%
*Age*		
18–35 y	286	14.3%
36–64 y	1165	58.2%
65–74 y	336	16.8%
75 y or older	215	10.7%
No data	2	–
*Household net equivalent income*		
Less than € 1000	185	13.6%
€ 1000–1499	231	17.0%
€ 1500–1999	400	29.4%
€ 2000–2999	332	24.4%
€ 3000–3999	111	8.2%
€ 4000 or more	100	7.4%
Don’t know / no data	645	–
*Highest educational qualification*		
No qualifications	4	0.2%
Elementary school / lower secondary school leaving certificate	266	13.5%
Intermediate school leaving certificate	391	19.9%
Vocational college diploma	208	10.6%
University entry qualification	1072	54.6%
Other educational certificate	24	1.2%
No details / still at school	39	–
*Prior medical experience (multiple answers possible)*		
Full-time in medical field	216	10.8%
Voluntary work in medical field	87	4.3%
Full-time in emergency services	44	2.2%
Voluntary work in emergency services	96	4.8%
Not in medical field	1589	79.3%
Don’t know / no details	7	–
*Experience with lay resuscitation*		
Has carried out a resuscitation	119	5.9%
Has not yet carried out a resuscitation	1411	94.1%

Defining age groups the present study used the classification followed in most industrialized countries [25] and formed two groups: interviewees up to and including age 64 (n = 1451) and those aged 65 or older (n = 551). As different levels of education might be a potential reason for different knowledge in the age-related analysis, the highest educational qualification according to the age of the sample is presented in S1 Table. Two participants were excluded from age-related analyses because they did not give their age. To allow more detailed analysis, this classification was supplemented by two additional groups of young participants (up to age 35) and very old participants (over 74).

### Registry data on bystander cardiopulmonary resuscitation

**Fig 1:**
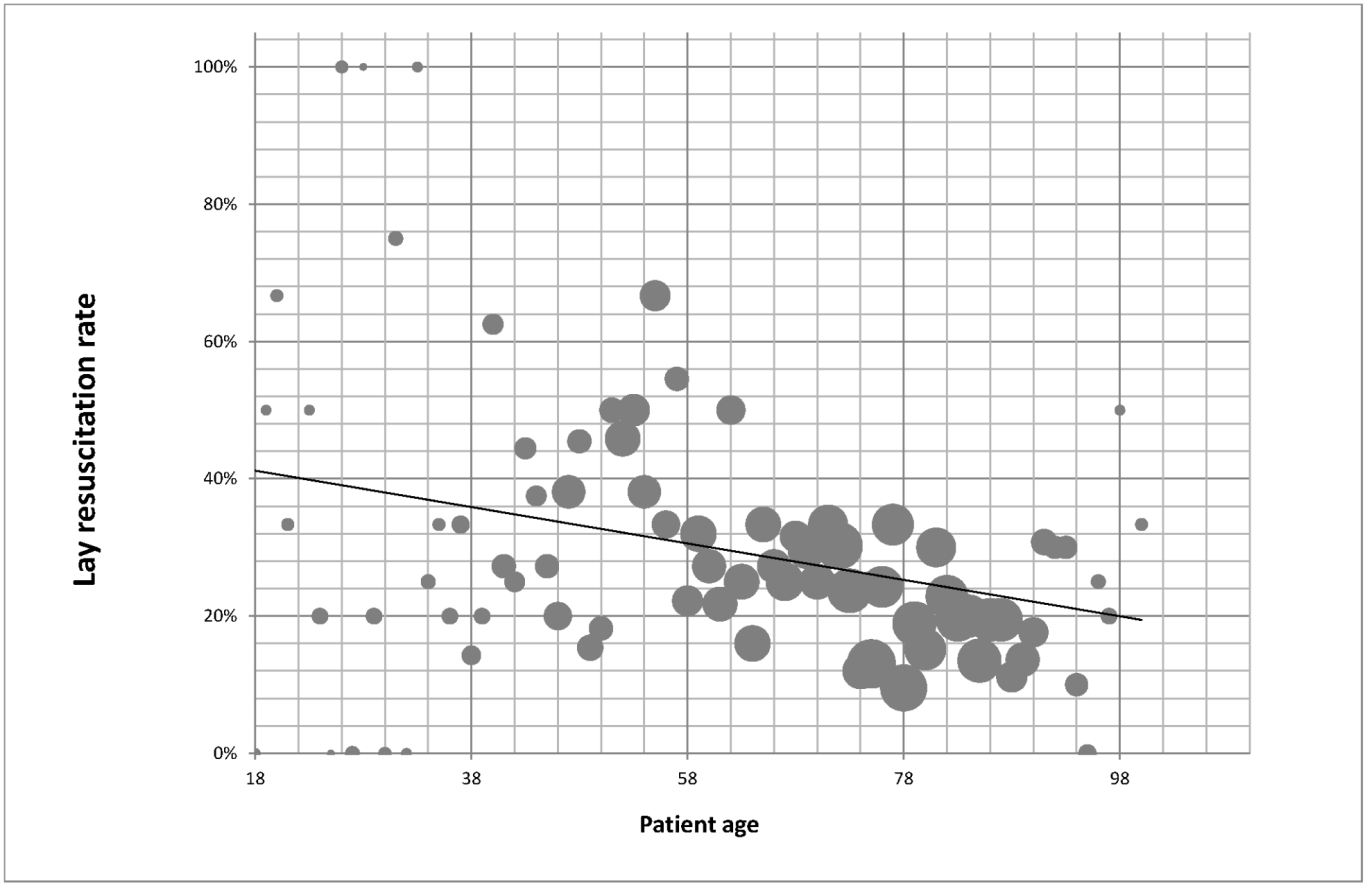
Lay resuscitation rate and patient age. There is a decline in the rate of lay resuscitation with increasing patient age. The size of the solid circles represents the numbers of resuscitations conducted in patients of each age.

A total of 1206 datasets from the resuscitation registry on out-of-hospital resuscitations were analysed. During this period, the bystander resuscitation rate was 29.9%. It was found that the probability of a patient being resuscitated by a bystander declined slightly with increasing patient age; the Pearson correlation coefficient was –0.052 (Fig 1). In the group of patients aged up to 35, 39.7% underwent resuscitation by bystanders; among patients aged 75 or over the rate was 21.8%. The cardiac arrest took place at home in 54.7% of those aged under 65, in comparison with 79.0% of those aged 65 or over (Table 3).

**Table 3:** Lay resuscitation rates and setting of cardiac arrest classified by age group.

Age	N	Lay CPR^a^		ROSC^b^		Home environment		Cardiac arrest observed	
		Absolute	Percentage	Absolute	Percentage	Absolute	Percentage	Absolute	Percentage
Total	1206	360	29.9%	551	45.7%	849	70.4%	657	54.5%
Up to 35 y	63	25	39.7%	30	47.6%	26	41.3%	28	44.4%
36–64 y	365	152	39.5%	177	48.5%	208	57.0%	190	52.1%
65–74 y	250	74	30.4%	128	51.2%	173	69.2%	159	63.6%
Over 74 y	528	115	21.8%	216	40.9%	442	83.7%	280	53.0%

The data presented here, taken from a total of 1361 out-of-hospital resuscitations, show that there is an association between increasing patient age and a declining rate of lay resuscitation

^a^CPR cardiopulmonary resuscitation;

^b^ROSC return of spontaneous circulation.

### Factual knowledge about lay resuscitation

After a brief case description of a patient with cardiac arrest had been given, the interviewees were asked about the correct emergency number, the steps to be taken, and the optimal frequency and compression depth for chest compressions. Correct answers for the emergency number were provided by 80.9% of the participants, and 63.6% chose ‘cardiac massage’ as the correct procedure. The correct compression depth and rate were only given by 58.3% and 16.4% of the interviewees, respectively.

When grouped by the age of the participants, a clearly lower level of information was found in older individuals. Interviewees aged over 65 had poorer results for all four of the information aspects mentioned above than younger participants did. 82.4% of interviewees under 65 years of age, knew the correct emergency number. In this group, 66.6% named cardiac massage as the relevant procedure in CA. Among older individuals these responses were only given by 75.1% and 49.5% (V = 0.082; P < 0.001 and V = 0.0157; P < 0.001).

**Fig 2:**
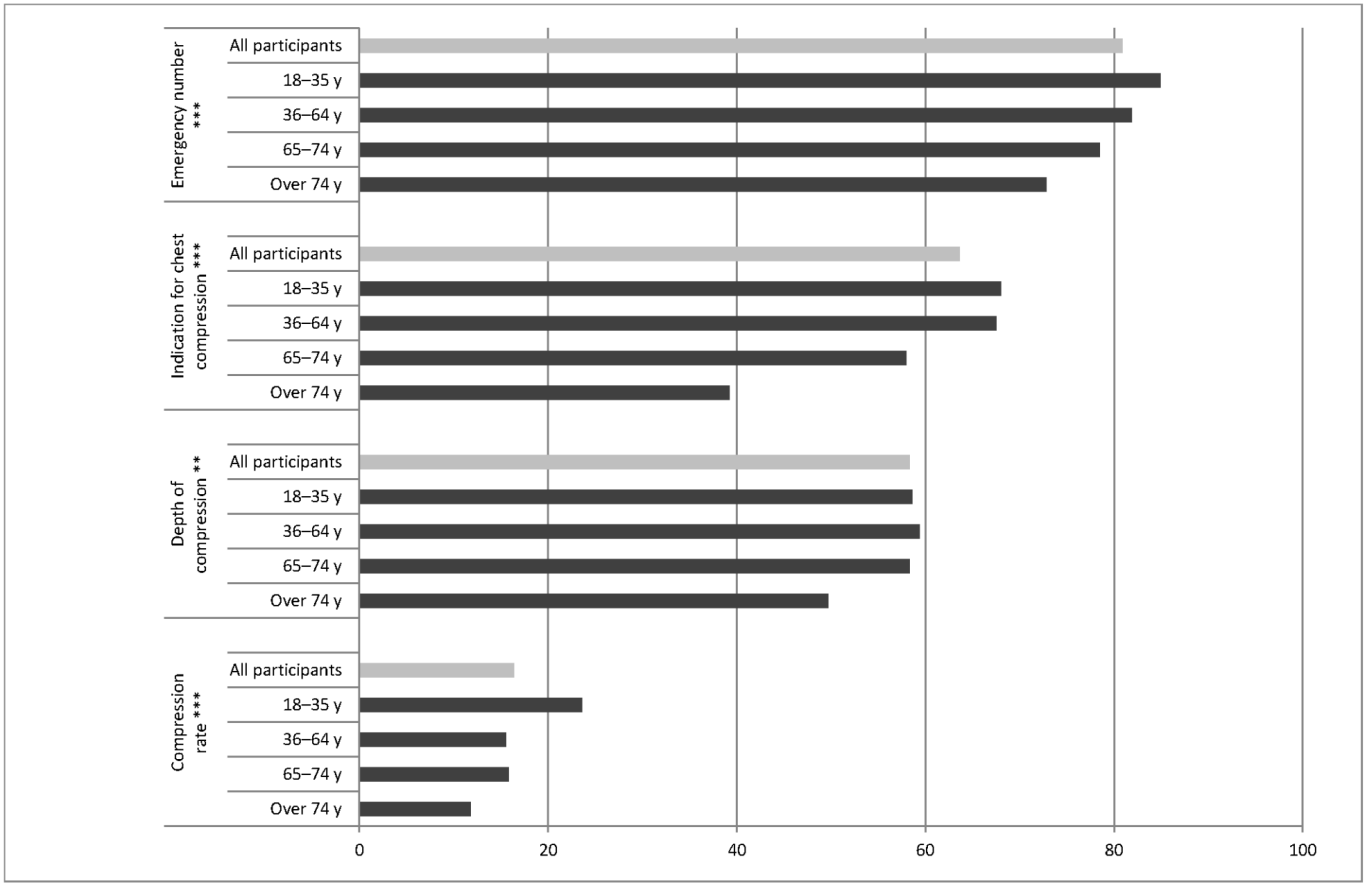
Information about cardiopulmonary resuscitation among the individuals participating in the questionnaire survey. Details are given as percentages for correct answers, both for the overall group (light grey bars) and divided into each age group (dark grey bars). *** Highly significant association, P < 0.005; ** very significant association, P < 0.01.

With a classification into four age groups (up to 35; 36–65; 66–75; and over 75), it was also found in nearly every area that the youngest participants showed the best results and the oldest ones the poorest (Fig 2).

Furthermore, older individuals know less about the use of an Automated External Defibrillator (AED). For example, 60,9% of interviewees aged over 64 fully or partly agreed to the statement that AEDs should only be used by medical staff. 61,1% in this age-group fully or partly agreed to the statement they would not use a defibrillator, because they would be afraid of making a mistake. This rejecting assessments of AED-use were given significantly less often among younger participants (40,1% and 44,7%, p<0.001).

### Participants’ self-assessment of their own abilities

The participants were requested to carry out a self-assessment of their own ability to carry out bystander resuscitation. 58.0% of the participants younger than 65 years were confident that they would detect cardiac arrest in comparison to 44.6% of the participants older than 65 years (V = 0.120; P < 0.001). Similarly, 62.7% of the interviewees younger than 65 were more certain to know what to do during CPR compared to 51.3% of the other group (V = 0.103; P < 0.001).

## Discussion

The present study investigated whether factual information about resuscitation differed among citizens in different age groups. Different knowledge about resuscitation in different age groups might be one of the reasons for decreased bystander CPR for older citizens.

The data show that the frequency of a layperson starting resuscitation measures was almost twice as high in patients under the age of 35 as it was in patients aged over 75. One can only speculate regarding the reasons for the difference. This is a limitation of our research, as it is impossible to identify a potential causal relationship by the study-design used. It is conceivable that potential providers of first aid regard death by sudden cardiac arrest as a ‘natural event’ more often in older patients. However, in view of the life expectancy of older people, this is a view that must be opposed [26]. Data regarding the return of spontaneous circulation (ROSC) in resuscitated individuals in the region studied also provide no foundation for the view that resuscitating older patients is not beneficial. An initial ROSC is achieved in 40.9% of cases even in patients aged over 74, not much less frequently than in the overall group of patients (45.7%).

Another possible reason for the lower bystander resuscitation rate in older patients might be an assumption that the quality of life is low in this group of patients following successful resuscitation and that resuscitation-attempts should therefore not be made for ethical reasons. However, research results showing the contrary have been published. Smith et al. [10], for example, showed using interviews with 697 resuscitated patients that their quality of life was good in the majority of cases of older patients.

Besides these general reasons for lower lay resuscitation rates in older patients, the bystanders who are present in each case may play a vital role. An analysis of the 1206 resuscitations included in this study showed that patients aged over 64 were found at home in the great majority of cases (79.8%). It is also known that spouses are the most frequent group of laypersons who observe cardiac arrest [15]. It is therefore likely that the potential providers of first aid in older patients with cardiac arrest are also themselves older persons. Swor et al. have shown that first-aid providers who start lay resuscitation are on average younger than those who do not initiate it [18]. Our results show that older individuals have less information about correct lay resuscitation procedure in several areas.

In addition, it is not only that older interviewees know less about carrying out lay resuscitation, but also that they regard themselves as less able to do so. It can be assumed that along with a poorer assessment of one’s own abilities, uncertainty that one will act correctly also increases. This is probably an additional factor contributing to the fact that older individuals start resuscitation measures less often, so that older patients are less likely to be resuscitated by laypersons.

The reason for older individuals knowing less about CPR measures remains unclear. A possible reason is poorer educational levels among the elderly (compare S1 Table). Furthermore, Papalexopoulou et al. have shown that retention of skills learned in courses is poorer among older participants [27]. However, it appears necessary to provide better training in resuscitation measures for older people. Such trainings might need to include the use of AEDs by layperson. This is of particular importance among the elderly, as retentions are higher and the use of an AED might be an option for someone being being unable to perform CPR due to physically impairment. From the authors’ point of view, targeted research needs to be carried out to assess how best to communicate the basic elements of resuscitation to older citizens, since little information is currently available about this.

### Limitations

#### Representativeness

As mentioned earlier, an attempt was made to make the study representative through a representative selection of phone numbers. The extent to which the actual participants reflected the total population was assessed using the characteristics of age distribution and sex distribution. As the results were satisfactory, the responses can be regarded as being largely representative of Münster. However, this also means that the results may not necessarily be transferable to other regions. This was a single-centre study, so that local influencing factors may limit its transferability. Furthermore, the uptake-rate of 29.4% bears the risk of a selection bias. Persons with particular interest in the subject might be overrepresented. Our results have to be interpreted against this background.

#### Discrepancy between questionnaire responses and action

It is generally difficult to draw conclusions from questionnaire responses about the actual way in which interviewees are likely to act in reality. In addition, the quality of lay resuscitation plays a decisive role in whether or not it is successful. Herlitz et al. have shown, for example, that positive outcomes are nearly twice as frequent if the person providing first aid is not a medical layperson but has relevant prior experience [28]. However, the quality of actual lay resuscitation cannot generally be assessed using questionnaires.

#### Limitations due to study-design

We conducted a study based on 2004 interviews and the retrospective analysis of resuscitation cases documented in the German Resuscitation Registry. This study gives deeper insight into the varying knowledge and self-confidence regarding CPR between younger and older individuals. However, due to the study design it is impossible to verify causal connections. The authors assume, that the lower rates of bystander CPR seen in the retrospective analysis are a result of older potential bystanders’ lower level of knowledge and self-confidence demonstrated in the interviews. However, this potential causal connection cannot be proofed by this study’s design.

## Conclusion

The probability that patients who require resuscitation will receive it from laypersons before the emergency services arrive declines with increasing patient age. One possible reason for this may be that potential providers of first aid in this group are themselves also older. This study shows that people over the age of 65 have less knowledge about resuscitation. At the same time people older than 65 have lower confidence in their ability to apply resuscitation than younger people have. Additional research should be carried out on this topic and appropriate publicity campaigns should be started if needed.

## Supporting information

S1 Table Highest educational qualification according to the age of the sample. (DOCX)

S2 Table Original data of the survey. Presented are the raw data underlying the findings presented. The column headings refer to the alphanumerical codes of the questions as shown in S1 and S2 Text.
(XLS)

S1 Text German questionnaire. The document S1 Text contains the surveys’ questionnaire in its original German version.
(DOCX)

S2 Text English questionnaire. The document S2 Text contains the surveys’ questionnaire translated into English.
(DOCX)
