# Supplementary material for: Senior citizens as rescuers: Is reduced knowledge the reason for omitted lay-resuscitation-attempts? Results from a representative survey with 2004 interviews
Source: PLoS One. 2017 Jun 12;12(6):e0178938. doi: 10.1371/journal.pone.0178938 (PMC5467835; doi:10.1371/journal.pone.0178938)
Supplement: S1 Table — (DOCX) [file pone.0178938.s001.docx]

**Table S1** Highest educational qualification according to the age of the sample.

|  | Total | | Absolute <65 years | Relative <65 years | Absolute >65 years | Relative >65 years |
| --- | --- | --- | --- | --- | --- | --- |
| No qualifications | | 4 | 2 | 0,13% | 2 | 0,39% |
| Elementary school / lower secondary school | | 266 | 125 | 8,41% | 140 | 27,18% |
| Intermediate school leaving certificate | | 391 | 261 | 17,55% | 130 | 25,24% |
| Vocational college diploma | | 208 | 169 | 11,37% | 39 | 7,57% |
| University entry qualification | | 1072 | 885 | 59,52% | 186 | 36,12% |
| Other educational certificate | | 24 | 11 | 0,74% | 13 | 2,52% |
| Still at school | | 22 | 17 | 1,14% | 5 | 0,97% |
| No Answer | | 17 | 17 | 1,14% | 0 | 0,00% |
| Sum | | 2002 | 1487 | 100,00% | 515 | 100,00% |
