# Supplementary material for: Senior citizens as rescuers: Is reduced knowledge the reason for omitted lay-resuscitation-attempts? Results from a representative survey with 2004 interviews
Source: PLoS One. 2017 Jun 12;12(6):e0178938. doi: 10.1371/journal.pone.0178938 (PMC5467835; doi:10.1371/journal.pone.0178938)
Supplement: S1 Text — The document S1 Text contains the surveys’ questionnaire in its original German version. (DOCX) [file pone.0178938.s003.docx]

| Nr. | Item | weiter mit |
| --- | --- | --- |
| **A** | **Eigene Erfahrung Erste Hilfe und Wiederbelebung** |  |
| A1 | Zunächst würde ich gerne von Ihnen wissen, ob Sie selber schon einmal bei einem medizinischem Notfall oder einem Unfall Erste Hilfe geleistet haben.  ja [ 1 ]  nein [ 2 ]  *(nicht vorlesen)* weiß nicht [ 98 ] | 🡺B1  🡺B1 |
| A2  A2a  A2b  A2c  A2d  A2e  A2f  A2g  A2z | In welcher Form haben Sie Erste Hilfe geleistet?  Bitte geben Sie an, welche der folgenden Maßnahmen Sie schon einmal angewendet haben.  *(Mehrfachantworten möglich)*  Hilfe gerufen, z.B. den Notarzt [ 1 ]  Unfallstelle gesichert [ 2 ]  Verletzungen versorgt [ 3 ]  Herzdruckmassage ohne Mund-zu-Mund-Beatmung [ 4 ]  Herzdruckmassage mit Mund-zu-Mund-Beatmung [ 5 ]  stabile Seitenlage [ 6 ]  sonstige erste Hilfe Maßnahmen [ 7 ]  *(nicht vorlesen)* weiß nicht [ 98 ] |  |
| A3 | Wenn Sie einmal an die letzte Situation denken, bei der Sie Erste Hilfe geleistet haben:  Wie sicher waren Sie sich, dass Sie in dieser Situation richtig gehandelt haben?  Bitte antworten Sie für diese Frage anhand einer Skala von 1 bis 6. Der Wert 1 bedeutet „sehr sicher“, der Wert 6 „völlig unsicher“. Mit den Werten dazwischen können Sie Ihre Meinung abstufen.  1 sehr sicher [ 1 ]  2 [ 2 ]  3 [ 3 ]  4 [ 4 ]  5 [ 5 ]  6 völlig unsicher [ 6 ]  *(nicht vorlesen)* weiß nicht [ 98 ] |  |
| **B** | **Kenntnisstand Wiederbelebungsmaßnahmen** |  |
| B1  B2  B3  B4  B5  B6 | Kommen wir nun zu Wiederbelebungsmaßnahmen bei Herzstillstand allgemein. Wir würden gerne wissen, wie der Kenntnisstand dazu in der Bevölkerung ist.  Bei welchen der folgenden Symptomen, die eine Person aufweist, ist es Ihrer Meinung nach sinnvoll, eine Herzdruckmassage durchzuführen?  Bitte geben Sie jeweils an, ob Sie es für sehr sinnvoll, weniger sinnvoll oder nicht sinnvoll halten.   \|  \| sehr sinnvoll \| weniger sinnvoll \| nicht sinnvoll \| weiß nicht \| \| --- \| --- \| --- \| --- \| --- \| \| keine Reaktion auf Ansprache und Schütteln \| [ 1 ] \| [ 2 ] \| [ 3 ] \| [ 98 ] \| \| keine normale Atmung, z.B. nach Luft schnappen \| [ 1 ] \| [ 2 ] \| [ 3 ] \| [ 98 ] \| \| gar keine Atmung wahrnehmbar \| [ 1 ] \| [ 2 ] \| [ 3 ] \| [ 98 ] \| \| kein Pulsschlag fühlbar \| [ 1 ] \| [ 2 ] \| [ 3 ] \| [ 98 ] \| \| Bewusstlosigkeit aber normale Atmung \| [ 1 ] \| [ 2 ] \| [ 3 ] \| [ 98 ] \| \| bläuliche Verfärbung der Haut \| [ 1 ] \| [ 2 ] \| [ 3 ] \| [ 98 ] \| |  |
| B7 | Welche Telefonnummer rufen Sie an, wenn jemand in Ihrer Umgebung plötzlich zusammenbricht und ärztliche Hilfe benötigt?  112 [ 1 ] 110 [ 2 ]  die Nummer Ihres Hausarztes [ 3 ]  die Nummer des kassenärztlichen Notdienstes [ 4 ]  *(nicht vorlesen)* 112 oder 110 [ 5 ]  *(nicht vorlesen)* eine andere Nummer [ 6 ]  *(nicht vorlesen)* weiß nicht [ 98 ] |  |
| B8 | Nehmen wir einmal an, bei einer Person wurde einen Herzstillstand festgestellt und der Notarzt wurde gerufen. Welche erste Hilfe Maßnahme sollte nun als Erstes durchführt werden: Eine Herzdruckmassage, eine Mund-zu-Mund-Beatmung oder sollte die Person in die stabile Seitenlage gebracht werden?  *(nicht vorlesen, Mehrfachantworten möglich)*  Herzdruckmassage [ 1 ]  Mund-zu-Mund-Beatmung [ 2 ]  Stabile Seitenlage [ 3 ]  nichts von alledem [ 4 ]  sonstiges [ 5 ]  weiß nicht [ 98 ] |  |
| B9 | Bei einer Herzdruckmassage wissen viele Menschen nicht genau, wie häufig man pro Minute auf den Brustkorb drücken muss.  Was meinen Sie, wie häufig sollte man bei einer Herzdruckmassage pro Minute drücken?  10 bis 40 Mal pro Minute [ 1 ]  60 bis 80 Mal pro Minute [ 2 ]  100 bis 120 Mal pro Minute [ 3 ]  140 bis 160 Mal pro Minute [ 4 ]  180 bis 200 Mal pro Minute [ 5 ]  *(nicht vorlesen)* weiß nicht [ 98 ] |  |
| B10 | Was meinen Sie, wie kräftig sollte man bei einer Herzdruckmassage drücken?  A. nur vorsichtig wie bei einer Massage [ 1 ]  B. so kräftig, dass der Brustkorb leicht zusammengedrückt   wird [ 2 ]  C. so kräftig, dass der Brustkorb mehrere Zentimeter  zusammengedrückt wird [ 3 ]  *(nicht vorlesen)* weiß nicht [ 98 ] |  |
| B11 | Wann sollte man Ihrer Meinung nach eine Herzdruckmassage beenden? Ich lese Ihnen nun zwei Aussagen vor und Sie sagen mir bitte, welche Aussage Ihrer Meinung nach eher zutrifft.  *(Int.: Die Antwort „bis die Person reagiert“ ist zwar richtig, bitte dennoch auf eine der beiden Aussagen festlegen lassen.)*  A. maximal 10 Minuten, weil danach keine  lebenserhaltende Wirkung mehr vorhanden ist. [ 1 ]  B. bis der Notarzt eintrifft, egal wie lange es dauert. [ 2 ]  *(nicht vorlesen)* weiß nicht [98 ] |  |
| B12  B13  B14  B15 | Ich nenne Ihnen nun einige Aussagen. Bitte antworten Sie jeweils anhand der Skala von 1 bis 6 in wie weit Sie der Aussage zustimmen. Der Wert 1 bedeutet „stimme voll und ganz zu“, der Wert 6 „stimme überhaupt nicht zu“. Mit den Werten dazwischen können Sie Ihre Meinung abstufen.   \|  \| stimme voll und ganz zu \|  \|  \|  \|  \| stimme überhaupt nicht zu \| weiß nicht \| \| --- \| --- \| --- \| --- \| --- \| --- \| --- \| --- \| \| Ich bin mir sicher, dass ich einen Herzstillstand sofort erkennen würde. \| [ 1 ] \| [ 2 ] \| [ 3 ] \| [ 4 ] \| [ 5 ] \| [ 6 ] \| [ 98 ] \| \| Ich weiß genau, was bei einer Herzdruckmassage zu beachten ist. \| [ 1 ] \| [ 2 ] \| [ 3 ] \| [ 4 ] \| [ 5 ] \| [ 6 ] \| [ 98 ] \| \| Bei einem Herzstillstand würde ich zunächst abwarten, ob jemand anderes eine Herzdruckmassage durchführt. \| [ 1 ] \| [ 2 ] \| [ 3 ] \| [ 4 ] \| [ 5 ] \| [ 6 ] \| [ 98 ] \| \| Wenn ich eine Herzdruckmassage durchführe, erhöhe ich die Wahrscheinlichkeit einer vollständigen Genesung entscheidend. \| [ 1 ] \| [ 2 ] \| [ 3 ] \| [ 4 ] \| [ 5 ] \| [ 6 ] \| [ 98 ] \| |  |
| **C** | **Anwendungsbereitschaft Wiederbelebungsmaßnahmen** |  |
| C1 | Viele Personen zögern im Ernstfall bei der Anwendung von Wiederbelebungsmaßnahmen. Wie schätzen Sie sich ein: Würden Sie im Notfall eine Wiederbelebungsmaßnahme beginnen?  ja, auf jeden Fall [ 1 ]  ja, aber nur wenn ich alleine bin [ 2 ]  nein, wahrscheinlich nicht [ 3 ]  nein, sicher nicht [ 4 ]  *(nicht vorlesen)* weiß nicht [ 98 ] | 🡺C8  🡺C8  🡺C2  🡺C2  🡺C8 |
| C2  C3  C4  C5  C6  C7 | Warum würden Sie nicht mit einer Wiederbelebungsmaßnahme beginnen?  Ich lese Ihnen nun einige wichtige Gründe dafür vor. Bitte sagen Sie mir jeweils, wie sehr diese auf Sie zutreffen.  Bitte antworten Sie wieder anhand einer Skala von 1 bis 6. Der Wert 1 bedeutet „stimme voll und ganz zu“, der Wert 6 „stimme überhaupt nicht zu“.   \|  \| stimme voll und ganz zu \|  \|  \|  \|  \| stimme überhaupt nicht zu \| weiß nicht \| \| --- \| --- \| --- \| --- \| --- \| --- \| --- \| --- \| \| Ich bin nicht kräftig genug für eine Herzdruckmassage. \| [ 1 ] \| [ 2 ] \| [ 3 ] \| [ 4 ] \| [ 5 ] \| [ 6 ] \| [ 98 ] \| \| Ich habe Angst, dass ich mich bei der Hilfeleistung mit einer Krankheit anstecke \| [ 1 ] \| [ 2 ] \| [ 3 ] \| [ 4 ] \| [ 5 ] \| [ 6 ] \| [ 98 ] \| \| Ich habe Angst, dass ich etwas falsch mache. \| [ 1 ] \| [ 2 ] \| [ 3 ] \| [ 4 ] \| [ 5 ] \| [ 6 ] \| [ 98 ] \| \| Ich habe Angst, dass ich bei einem Fehler verklagt werde. \| [ 1 ] \| [ 2 ] \| [ 3 ] \| [ 4 ] \| [ 5 ] \| [ 6 ] \| [ 98 ] \| \| Ich weiß nicht genau, was ich bei einer Herzdruckmassage machen sollte \| [ 1 ] \| [ 2 ] \| [ 3 ] \| [ 4 ] \| [ 5 ] \| [ 6 ] \| [ 98 ] \| \| Ich wäre bei einem solchen Notfall zu gestresst, um eine Herzdruckmassage durchzuführen. \| [ 1 ] \| [ 2 ] \| [ 3 ] \| [ 4 ] \| [ 5 ] \| [ 6 ] \| [ 98 ] \| |  |
| C8 | Das Personal, das Notrufe entgegennimmt, kann Sie telefonisch bei der Durchführung von Wiederbelebungsmaßnahmen anleiten. Bitte schätzen Sie auf einer Skala von 1 bis 6 ein, wie hilfreich eine solche telefonische Anleitung für Sie wäre.  Der Wert 1 bedeutet sehr hilfreich und der Wert 6 überhaupt nicht hilfreich.  1 sehr hilfreich [ 1 ]  2 [ 2 ]  3 [ 3 ]  4 [ 4 ]  5 [ 5 ]  6 überhaupt nicht hilfreich [ 6 ]  *(nicht vorlesen)* weiß nicht [ 98 ] | 🡺C10  🡺C10  🡺C10  🡺C10 |
| C9 | Warum wäre eine telefonische Anleitung für Sie weniger oder nicht hilfreich?  Bitte sagen Sie mir, welcher der folgenden Gründe am ehesten auf Sie zutrifft.  A. ich weiß, was zu tun ist [ 1 ]  B. ich würde mir auch mit dieser Hilfe keine   Wiederbelebung zutrauen [ 2 ]  C. ich halte eine telefonische Beratung   für viel zu umständlich [ 3 ]  *(nicht vorlesen)* anderer Grund [ 4 ]  *(nicht vorlesen)* weiß nicht [ 98 ] |  |
| C10 | In den letzten Jahren stehen vermehrt Defibrillatoren an öffentlichen Orten zur Verfügung. Hierbei handelt es sich um Geräte, die mit Elektroschocks die Wiederbelebungsmaßnahme automatisch unterstützt.  *(Int. Gemeint sind sog. AED = Automatisierter Externer Defibrillator)*  Haben Sie schon einmal an einem Training zum Umgang mit einem solchen Gerät teilgenommen?  ja [ 1 ]  nein [ 2 ]  *(nicht vorlesen)* weiß nicht [ 98 ] |  |
| C11  C12  C13  C14 | Welcher Aussage zu öffentlich zugänglichen Defibrillatoren stimmen Sie eher zu?  Ich lese Ihnen nun vier Aussage zu diesem Thema vor. Bitte sagen Sie mir jeweils, in wie weit Sie den Aussage zustimmen bzw. nicht zustimmen. Der Wert 1 bedeutet „stimme voll und ganz zu“, der Wert 6 „stimme überhaupt nicht zu“.   \|  \| stimme voll und ganz zu \|  \|  \|  \|  \| stimme überhaupt nicht zu \| weiß nicht \| \| --- \| --- \| --- \| --- \| --- \| --- \| --- \| --- \| \| Defibrillatoren sollten nur von medizinisch geschultem Fachpersonal benutzt werden \| [ 1 ] \| [ 2 ] \| [ 3 ] \| [ 4 ] \| [ 5 ] \| [ 6 ] \| [ 98 ] \| \| Defibrillatoren erhöhen die Überlebenschancen bei einem Herzstillstand erheblich \| [ 1 ] \| [ 2 ] \| [ 3 ] \| [ 4 ] \| [ 5 ] \| [ 6 ] \| [ 98 ] \| \| Ich würde einen Defibrillator nicht benutzen, weil ich Angst hätte, etwas falsch zu machen \| [ 1 ] \| [ 2 ] \| [ 3 ] \| [ 4 ] \| [ 5 ] \| [ 6 ] \| [ 98 ] \| \| Ich würde einen Defibrillator nicht benutzen, weil ich Angst hätte, mich damit selber zu verletzten \| [ 1 ] \| [ 2 ] \| [ 3 ] \| [ 4 ] \| [ 5 ] \| [ 6 ] \| [ 98 ] \| |  |
| C15 | Studien haben gezeigt, dass es bei einem Laien völlig ausreichend ist, wenn er bei einer Wiederbelebungsmaßnahme nur eine Herzdruckmassage und keine Mund-zu-Mund-Beatmung durchführt.  Glauben Sie, dass Sie eher bereit sind eine Wiederbelebungsmaßnahme durchzuführen, wenn Sie keine Mund-zu-Mund-Beatmung anwenden müssen?  ja, ich wäre eher bereit zu helfen [ 1 ]  nein, es hätte keinen Einfluss auf meine Bereitschaft [ 2 ]  *(nicht vorlesen)* weiß nicht [ 98 ] |  |
| **D** | **Quellen Informationen zur Wiederbelebung allgemein** |  |
| D1 | Haben Sie schon einmal an einem Erste-Hilfe-Kurs teilgenommen?  ja [ 1 ]  nein [ 2 ]  *(nicht vorlesen)* mehrfach teilgenommen [ 3 ]  *(nicht vorlesen)* weiß nicht [ 98 ] | 🡺D2  🡺D5  🡺D2  🡺D5 |
| D2 | Wie lange liegt ihre letzte Teilnahme an einem Erste-Hilfe-Kurs zurück?  bis zu einem Jahr [ 1 ]  bis zu zwei Jahren [ 2 ]  bis zu fünf Jahren [ 3 ]  bis zu zehn Jahren [ 4 ]  zehn Jahre und mehr [ 5 ]  *(nicht vorlesen)* weiß nicht [ 98 ] | 🡺D4  🡺D4  🡺D4  🡺D3  🡺D3  🡺D4 |
| D3  D3a  D3b  D3c  D3d  D3e  D3f  D3g  D3z | Warum haben Sie schon so lange nicht mehr an einem Erste-Hilfe-Kurs teilgenommen?  Ich nenne Ihnen nun einige mögliche Gründe und Sie sagen mit bitte immer jeweils, ob der Grund für Sie zutrifft.  *(Int.: Items einzeln vorlesen, Mehrfachantworten sind möglich)*  fehlende Informationen über Angebote [ 1 ]  zu hohe Kosten für die Teilnahme [ 2 ]  mangelnde Zeit [ 3 ]  mangelnde Motivation [ 4 ]  ausreichende Erste-Hilfe-Kenntnisse vorhanden [ 5 ]  ich bin dafür zu alt [ 6 ]  *(nicht vorlesen)* sonstige Gründe [ 6 ]  *(nicht vorlesen)* weiß nicht [ 98 ] |  |
| D4  D4a  D4b  D4c  D4d  D4e  D4f  D4g  D4z | Es gibt verschiedene Gründe, warum man einen Erste-Hilfe-Kurs besucht. Ich lese Ihnen nun einige davon vor. Bitte geben Sie jeweils an, ob diese für Ihre letzte Teilnahme ausschlaggebend waren.  (Int.: Items einzeln vorlesen, Mehrfachantworten sind möglich)  für den Erwerb des Führerscheins [ 1 ]  aufgrund meiner Funktion als Ersthelfer am Arbeitsplatz. [ 2 ]  aufgrund einer Tätigkeit in der Freizeit (Ehrenamt, Sport, Hobby, usw.) [ 3 ]  weil ich bei einem Notfall nicht wusste, was zu tun war [ 4 ]  weil ich ganz allgemein das Gefühl hatte, dass eine Kursteilnahme sinnvoll ist [ 5 ]  aufgrund eines Angebots in der Schule [ 6 ]  (nicht vorlesen) sonstige Gründe [ 6 ]  (nicht vorlesen) weiß nicht [ 98 ] | 🡺D6  🡺D6  🡺D6  🡺D6  🡺D6  🡺D6  🡺D6  🡺D6 |
| D5  D5a  D5b  D5c  D5d  D5e  D5f  D5z | Warum haben Sie noch nie an einem Erste-Hilfe-Kurs teilgenommen?  *(Int.: Mehrfachantworten sind möglich)*  fehlende Informationen über Angebote [ 1 ]  zu hohe Kosten für die Teilnahme [ 2 ]  mangelnde Zeit [ 3 ]  mangelnde Motivation [ 4 ]  ausreichende Erste-Hilfe-Kenntnisse vorhanden [ 5 ]  *(nicht vorlesen)* sonstige Gründe [ 6 ]  *(nicht vorlesen)* weiß nicht [ 98 ] |  |
| D6 | Es gibt auch sehr kurze Kurse, die ausschließlich über Wiederbelebungsmaßnahmen bei einem Herzstillstand informieren.  Haben Sie schon einmal an einem solchen Kurs teilgenommen?  ja [ 1 ]  nein [ 2 ]  *(nicht vorlesen)* mehrfach teilgenommen [ 3 ]  *(nicht vorlesen)* weiß nicht [ 98 ] |  |
| D7  D8  D9 | Im Folgenden würde ich gerne wissen, wie wahrscheinlich es ist, dass Sie in den nächsten zwölf Monaten an einen Erste-Hilfe-Kurs teilnehmen werden.  Ich lese Ihnen nun drei verschiedene Angebote vor und Sie sagen mir bitte jeweils auf der Skala von 1 bis 6, wie wahrscheinlich es, dass Sie einen solchen Kurs belegen werden. Der Wert 1 bedeutet „sehr wahrscheinlich“, der Wert 6 „sehr unwahrscheinlich“.   \|  \| sehr  wahrscheinlich \| \| \| sehr  unwahr-scheinlich \| \| \| spontan: würde keinen Kurs besuchen \| weiß nicht \| \| --- \| --- \| --- \| --- \| --- \| --- \| --- \| --- \| --- \| \| einen Erste-Hilfe-Lehrgang mit 16 Unterrichtsstunden \| [ 1 ] \| [ 2 ] \| [ 3 ] \| [ 4 ] \| [ 5 ] \| [ 6 ] \| [ 7 ] \| [ 98 ] \| \| einen Erste-Hilfe-Wiederholungskurs mit 8 Unterrichtsstunden \| [ 1 ] \| [ 2 ] \| [ 3 ] \| [ 4 ] \| [ 5 ] \| [ 6 ] \| [ 7 ] \| [ 98 ] \| \| einen Wiederbelebungs-Kurs mit 2 Unterrichtsstunden \| [ 1 ] \| [ 2 ] \| [ 3 ] \| [ 4 ] \| [ 5 ] \| [ 6 ] \| [ 7 ] \| [ 98 ] \| | 7 = D10 |
| D10  D10a  D10b  D10c  D10d  D10e  D10f  D10g  D10z | Neben Erste-Hilfe-Kursen gibt es noch weitere Informationsquellen über Wiederbelebungsmaßnahmen bei einem Herzstillstand. Ich lese Ihnen nun einige davon vor. Bitte geben Sie jeweils an, ob Sie in den letzten 12 Monaten diese wahrgenommen haben.  *(Mehrfachantworten sind möglich)*  Broschüren und Faltblätter [ 1 ]  Berichte in Zeitungen und Zeitschriften [ 2 ]  Beiträge im Radio [ 3 ]  Beiträge im Fernsehen. [ 4 ]  Internetseiten [ 5 ]  Plakate [ 6 ]  Gespräche mit Freunde und Bekannten [ 7 ]  *(nicht vorlesen)* sonstige Quellen [ 8 ]  *(nicht vorlesen)* weiß nicht [ 98 ] | 🡺 F1  🡺 F1  🡺 F1  🡺 F1  🡺 D11  🡺 F1  🡺 F1  🡺 F1  🡺 F1 |
| D11  D11a  D11b  D11c  D11c | Hat es sich bei den Internetseiten um die folgenden Seiten gehandelt?  *(Mehrfachantworten sind möglich)*  www.einlebenretten.de [ 1 ]  www.100-pro-reanimation.de [ 2 ]  eine andere Seite [ 3 ]  *(nicht vorlesen)* weiß nicht [ 98 ] |  |
| **E** | **Wahrnehmung Woche der Wiederbelebung (nur zweite Befragung)** |  |
| E1 | Die Fragen folgen in der 2. Befragung |  |
| **F** | **Demographie** |  |
| F1 | Abschließend möchte ich Sie noch um einige Angaben zu Ihrer Person bitten:  Wie alt sind Sie? ______ Jahre  *(nicht vorlesen)* keine Antwort [ 99 ] |  |
| F2 | Welchen Familienstand haben Sie?  *(nicht vorlesen)* verheiratet/ eingetragene Lebensgemeinschaft [ 1 ]  ledig [ 2 ]  geschieden [ 3 ]  verwitwet [ 4 ]  anderer Familienstand [ 5 ]  keine Antwort [ 99 ] |  |
| F3 | Wie viele Personen leben in Ihrem Haushalt – Sie selbst mitgerechnet?  *(nicht vorlesen)* eine Person [ 1 ]  zwei Personen [ 2 ]  drei Personen [ 3 ]  vier Personen [ 4 ]  fünf Personen [ 5 ]  sechs Personen [ 6 ]  mehr als sechs Personen [ 7 ]  weiß nicht [ 98 ]  keine Antwort [ 99 ] | 🡺 D10 |
| F4  F4a  F4b  F4c  F4d  F4e  F4z | Mit welchen Personen leben Sie in Ihrem Haushalt zusammen?  **(Int.: Mehrfachantworten möglich)**  Ehe-/ LebenspartnerIn [ 1 ]  Kind/ Kinder [ 2 ]  andere Familienangehörige (Eltern, Geschwister, etc.) [ 3 ]  Wohngemeinschaft [ 4 ]  sonstige Personen [ 5 ]  *(nicht vorlesen)* keine Antwort [ 99 ] | Wenn F4_2≠1  🡺 F5 |
| F5 | Wie viele der Kinder sind jünger als 14 Jahre?    _______ Kind/er  *(nicht vorlesen)* keine Antwort [ 99 ] |  |
| F6 | Was ist Ihr höchster Schulabschluss?  *(nicht vorlesen)* kein Abschluss [ 1 ]  Volks- / Hauptschulabschluss [ 2 ]  Realschulabschluss (Mittlere Reife) [ 3 ]  Fachhochschulreife [ 4 ]  allgemeine Hochschulreife (Abitur) [ 5 ]  anderer Abschluss [ 6 ]  noch Schüler [ 7 ]  keine Antwort [ 99 ] | 🡺F8 |
| F7 | Welchen beruflichen Ausbildungsabschluss haben Sie?  Hochschulabschluss [ 1 ]  Fachhochschulabschluss (auch Ingenieurschule) [ 2 ]  Meister-, Techniker- oder gleichwertiger Berufs-Fachschulabschluss [ 3 ]  gewerbliche oder landwirtschaftliche Lehre [ 4 ]  kaufmännische Lehre [ 5 ]  (noch) kein Abschluss [ 6 ]  *(nicht vorlesen)* Berufliches Praktikum, Volontariat [ 7 ]  Teilfacharbeiterabschluss [ 8 ]  Beruflich-betriebliche Anlernzeit mit Abschlusszeugnis [ 9 ]  anderer beruflicher Abschluss [ 10 ]  weiß nicht [ 98 ]  keine Antwort [ 99 ] |  |
| F8  F8a  F8b  F8c  F8d  F8e  F8z | Sind oder waren Sie haupt- oder ehrenamtlich im medizinischen oder rettungsdienstlichen Bereich tätig?  *(Mehrfachantworten sind möglich, nicht vorlesen)*  hauptamtlich im medizinischen Bereich tätig [ 1 ]  ehrenamtlich im medizinischen Bereich tätig [ 2 ]  hauptamtlich im rettungsdienstlichen Bereich tätig [ 3 ]  ehrenamtlich im rettungsdienstlichen Bereich tätig [ 4 ]  nicht im medizinischen Bereich tätig [ 5 ]    weiß nicht [ 98 ] |  |
| F9 | Wie hoch ist das monatliche Nettoeinkommen Ihres Haushalts? Ich meine damit die Summe, die nach Abzug der Steuern und Sozialversicherungsbeiträge übrig bleibt.  *(ungefähre Schätzung; bei WG-Bewohnern nur das eigene Einkommen)*  unter 1000 Euro [ 1 ]  1000 bis 2000 Euro [ 2 ]  2001 bis 3000 Euro [ 3 ]  3001 bis 4000 Euro [ 4 ]  4001 bis 5000 Euro [ 5 ]  mehr als 5000 Euro [ 6 ]  *(nicht vorlesen)* weiß nicht [ 98 ]  keine Antwort [ 99 ] |  |
|  | **ENDE**  **Haben Sie noch Fragen zu unserer Umfrage? Vielen Dank für das Gespräch.** |  |
| F10 | Geschlecht der/des Befragten  weiblich [ 1 ]  männlich [ 2 ] |  |
