# Supplementary material for: Senior citizens as rescuers: Is reduced knowledge the reason for omitted lay-resuscitation-attempts? Results from a representative survey with 2004 interviews
Source: PLoS One. 2017 Jun 12;12(6):e0178938. doi: 10.1371/journal.pone.0178938 (PMC5467835; doi:10.1371/journal.pone.0178938)
Supplement: S2 Text — The document S2 Text contains the surveys’ questionnaire translated into English. (DOCX) [file pone.0178938.s004.docx]

| Nr. | Item | Continue with |
| --- | --- | --- |
| **A** | **Experience in first aid and resuscitation** |  |
| A1 | First of all I would like to know, if you have ever had to give first-aid in case of an accident or medical emergency?  Yes [ 1 ]  No [ 2 ]  *(do not read out loud)* I don’t know [ 98 ] | 🡺B1  🡺B1 |
| A2  A2a  A2b  A2c  A2d  A2e  A2f  A2g  A2z | In which way did you provide first aid?  Please list, which of the following measure you took?  *(multiple response possible)*  Call for Help, f.e. emergency doctor [ 1 ]  Secure the site of the accident [ 2 ]  Treat an injury [ 3 ]  Resuscitation without mouth-to-mouth-ventilation [ 4 ]  Resuscitation with mouth-to-mouth-ventilation [ 5 ]  Recovery position [ 6 ]  Further first-aid measures [ 7 ]  *(do not read out loud)* I don’t know [ 98 ] |  |
| A3 | If you think back to the last situation you gave first-aid:  How sure were you of acting correctly in this situation?  Please respond in a scale from 1 to 6, in which 1 means „very sure“ and 6 „ very insecure“  1 very sure [ 1 ]  2 [ 2 ]  3 [ 3 ]  4 [ 4 ]  5 [ 5 ]  6 very insecure [ 6 ]  *(do not read out loud)* I don’t know [ 98 ] |  |
| **B** | **Level of Knowledge: Resuscitation** |  |
| B1  B2  B3  B4  B5  B6 | Let’s talk about first-aid in case of cardiac arrest. We would like to know the general knowledge of the population.  For which of the following symptoms do you think it necessary to start resuscitation?  Please specify if it is “very useful”, “less useful” or “not useful”.   \|  \| very  useful \| less  useful \| not  useful \| I don’t know \| \| --- \| --- \| --- \| --- \| --- \| \| no response to being talked to or shaken \| [ 1 ] \| [ 2 ] \| [ 3 ] \| [ 98 ] \| \| abnormal ventilation, f.e. gasping for air \| [ 1 ] \| [ 2 ] \| [ 3 ] \| [ 98 ] \| \| no breathing \| [ 1 ] \| [ 2 ] \| [ 3 ] \| [ 98 ] \| \| no pulse detectable \| [ 1 ] \| [ 2 ] \| [ 3 ] \| [ 98 ] \| \| unconscious but normal breathing \| [ 1 ] \| [ 2 ] \| [ 3 ] \| [ 98 ] \| \| bluish discoloration of the skin \| [ 1 ] \| [ 2 ] \| [ 3 ] \| [ 98 ] \| |  |
| B7 | Which number would you call, if someone nearby collapsed or needed medical help?  112 [ 1 ] 110 [ 2 ]  Number of your family doctor [ 3 ]  Number of “Kassenärztlicher Notdienst” (something like a public  insurance doctor on call) [ 4 ]  *(do not read out loud)* 112 or 110 [ 5 ]  *(do not read out loud)* another number [ 6 ]  *(do not read out loud)* I don’t know [ 98 ] |  |
| B8 | Let’s assume, there is a person with cardiac arrest and the ambulance service is called. Which of the following first-aid measures should be started with: chest compressions, mouth-to-mouth-ventilation or ley in recovery position?  *(do not read out loud, multiple response possible)*  Chest compressions [ 1 ]  Mouth-to-mouth-ventilation [ 2 ]  Recovery position [ 3 ]  None of the above [ 4 ]  Others [ 5 ]  I don’t know [ 98 ] |  |
| B9 | When it comes to resuscitation a lot of people do not know how many times per minute they have to perform chest compressions. What do you think? How often should you perform chest compression per minute?  10 to 40 times per minute [ 1 ]  60 to 80 times per minute [ 2 ]  100 to 120 times per minute [ 3 ]  140 to 160 times per minute [ 4 ]  180 to 200 Mal per minute [ 5 ]  *(do not read out loud)* I don’t know [ 98 ] |  |
| B10 | What do you think: how hard should you press in case of resuscitation?  A. careful, like a massage [ 1 ]  B. powerful, so that the chest is compressed a little bit [ 2 ]  C. powerful, so that the chest is compressed for a couple of  centimeters [ 3 ]  *(do not read out loud)* I don’t know [ 98 ] |  |
| B11 | When do you think you should terminate resuscitation? I will give you two statements and you tell me which one you feel is correct.    (Int: The answer „until the Person shows a reaction“ also is a correct answer, but they should decide for one of the two given statements.)  A. max. 10 minutes, because after that there is no life-sustaining effect anymore. [ 1 ]  B. until the ambulance arrives, no matter how long it takes. [ 2 ]  *(do not read out loud)* I don’t know [98 ] |  |
| B12  B13  B14  B15 | I will now read out a few statements for you. Please respond in a scale from 1 to 6, in which 1 means „I agree completely“ and 6 „ I disagree completely“. You can use the numbers in between to graduate your opinion.   \|  \| I agree  completely \|  \|  \|  \|  \| I  disagree  completely \| I don’t  know \| \| --- \| --- \| --- \| --- \| --- \| --- \| --- \| --- \| \| I am confident that I would detect a cardiac arrest in a timely manner. \| [ 1 ] \| [ 2 ] \| [ 3 ] \| [ 4 ] \| [ 5 ] \| [ 6 ] \| [ 98 ] \| \| I know what I have to do to perform resuscitation. \| [ 1 ] \| [ 2 ] \| [ 3 ] \| [ 4 ] \| [ 5 ] \| [ 6 ] \| [ 98 ] \| \| In case of a cardiac arrest I would wait and see if someone else will start resuscitation. \| [ 1 ] \| [ 2 ] \| [ 3 ] \| [ 4 ] \| [ 5 ] \| [ 6 ] \| [ 98 ] \| \| When I do resuscitation, I increase the probability of a complete recovery. \| [ 1 ] \| [ 2 ] \| [ 3 ] \| [ 4 ] \| [ 5 ] \| [ 6 ] \| [ 98 ] \| |  |
| **C** | **Willingness to do resuscitation** |  |
| C1 | A lot of people hesitate when it comes to a real case of need of resuscitation. How do you rate yourself: would you start resuscitation in a real situation?  Yes, of course [ 1 ]  Yes, but only if I am alone [ 2 ]  No, probably not [ 3 ]  No, I would not [ 4 ]  *(do not read out loud)* I don’t know [ 98 ] | 🡺C8  🡺C8  🡺C2  🡺C2  🡺C8 |
| C2  C3  C4  C5  C6  C7 | What could be reasons for you to not start with resuscitation?  I will now read out a few statements for you. Please respond in a scale from 1 to 6, in which 1 means „I agree completely“ and 6 „ I disagree completely“. You can use the numbers in between to graduate your opinion.   \|  \| I agree  completely \|  \|  \|  \|  \| I  disagree  completely \| I don’t  know \| \| --- \| --- \| --- \| --- \| --- \| --- \| --- \| --- \| \| I am not strong enough to perform resuscitation \| [ 1 ] \| [ 2 ] \| [ 3 ] \| [ 4 ] \| [ 5 ] \| [ 6 ] \| [ 98 ] \| \| I am afraid to infect myself during the resuscitation. \| [ 1 ] \| [ 2 ] \| [ 3 ] \| [ 4 ] \| [ 5 ] \| [ 6 ] \| [ 98 ] \| \| I am afraid to do something wrong. \| [ 1 ] \| [ 2 ] \| [ 3 ] \| [ 4 ] \| [ 5 ] \| [ 6 ] \| [ 98 ] \| \| I am afraid to get sued because of a mistake. \| [ 1 ] \| [ 2 ] \| [ 3 ] \| [ 4 ] \| [ 5 ] \| [ 6 ] \| [ 98 ] \| \| I am not sure how to perform a resuscitation. \| [ 1 ] \| [ 2 ] \| [ 3 ] \| [ 4 ] \| [ 5 ] \| [ 6 ] \| [ 98 ] \| \| In case of a real emergency I would be too nervous to perform resuscitation. \| [ 1 ] \| [ 2 ] \| [ 3 ] \| [ 4 ] \| [ 5 ] \| [ 6 ] \| [ 98 ] \| |  |
| C8 | The staff taking the emergency call can help you by giving instructions over the telephone throughout the entire resuscitation. Please estimate how helpful this would be for you on a scale from 1 to 6, in which 1 means „very helpful“ and 6 „not helpful“. You can use the numbers in between to graduate your opinion  1 very helpful [ 1 ]  2 [ 2 ]  3 [ 3 ]  4 [ 4 ]  5 [ 5 ]  6 not helpful [ 6 ]  *(do not read out loud)* I don’t know [ 98 ] | 🡺C10  🡺C10  🡺C10  🡺C10 |
| C9 | For what reasons are instructions over the telephone less or not helpful?  Please tell me, which reason represents your opinion the most.  A. I know what I have to do [ 1 ]  B. Not even with this help would I dare to resuscitate [ 2 ]  C. I think instructions over the telephone are a way to complicated [ 3 ]  *(do not read out loud)* other reasons [ 4 ]  *(do not read out loud)* I don’t know [ 98 ] |  |
| C10 | Within the last years more and more public places have been equipped with defibrillators. These defibrillators are able to support resuscitation automatically with electric shocks.  (Meant are AED = Automatic external defibrillator)  Did you ever complete a training to use such equipment?  Yes [ 1 ]  No [ 2 ]  *(do not read out loud)* I don’t know [ 98 ] |  |
| C11  C12  C13  C14 | Which statement about such public defibrillators do you most agree with?  I will now read out four statements. Please respond in a scale from 1 to 6, in which 1 means „I agree completely“ and 6 „ I disagree completely“. You can use the numbers in between to graduate your opinion.   \|  \| I agree  completely \|  \|  \|  \|  \| I disagree completely \| I don’t know \| \| --- \| --- \| --- \| --- \| --- \| --- \| --- \| --- \| \| Defibrillators should only be used by medical staff \| [ 1 ] \| [ 2 ] \| [ 3 ] \| [ 4 ] \| [ 5 ] \| [ 6 ] \| [ 98 ] \| \| Defibrillators increase the probability to survive in case of a cardiac arrest significantly \| [ 1 ] \| [ 2 ] \| [ 3 ] \| [ 4 ] \| [ 5 ] \| [ 6 ] \| [ 98 ] \| \| I wouldn’t use a defibrillator, because I am afraid of making a mistake \| [ 1 ] \| [ 2 ] \| [ 3 ] \| [ 4 ] \| [ 5 ] \| [ 6 ] \| [ 98 ] \| \| I wouldn’t use a defibrillator, because I am afraid to injure myself \| [ 1 ] \| [ 2 ] \| [ 3 ] \| [ 4 ] \| [ 5 ] \| [ 6 ] \| [ 98 ] \| |  |
| C15 | Research shows that it is sufficient for a non-professional to just perform chest pressure without mouth-to-mouth-ventilation in case of resuscitation.  Do you think, you would rather do resuscitation if you didn’t have to do mouth-to-mouth-ventilation?  Yes, I would rather do it [ 1 ]  No, it has no influence on my willingness to resuscitate [ 2 ]  *(do not read out loud)* I don’t know [ 98 ] |  |
| **D** | **Sources of information about resuscitation in general** |  |
| D1 | Did you ever participate in a first-aid-training?  Yes [ 1 ]  No [ 2 ]  *(do not read out loud)* Multiple times [ 3 ]  *(do not read out loud)* I don’t know [ 98 ] | 🡺D2  🡺D5  🡺D2  🡺D5 |
| D2 | How long has it been since you last participated in a first-aid-training?  Less than a year [ 1 ]  Less than two years [ 2 ]  Less than five years [ 3 ]  Less than ten years [ 4 ]  More than ten years [ 5 ]  *(do not read out loud)* I don’t know [ 98 ] | 🡺D4  🡺D4  🡺D4  🡺D3  🡺D3  🡺D4 |
| D3  D3a  D3b  D3c  D3d  D3e  D3f  D3g  D3z | Why was your last participation in a first-aid-training such a long time ago?  I will read out some possible reasons, please tell me, if a reason applies to you or not.  *(Int.: read items individually, multiple responses possible)*  Missing Information about first-aid-training [ 1 ]  Courses were too expensive [ 2 ]  I did not have time [ 3 ]  I did not have a motivation to participate [ 4 ]  I know enough about first-aid [ 5 ]  I am too old for something like that [ 6 ]  *(do not read out loud)* other reasons [ 6 ]  *(do not read out loud)* I don’t know [ 98 ] |  |
| D4  D4a  D4b  D4c  D4d  D4e  D4f  D4g  D4z | There are many reasons why someone could participate in a first-aid-training. I will read out some of them. Please tell me is the reason had an influence on your last participation.  *(Int.: read items particular, multiple response possible)*  To acquire your drivers license [ 1 ]  As a part of my role as first-aid responder at work [ 2 ]  Because of an activity in my free time (voluntary work, sport, hobby, etc.) [ 3 ]  Because I once did not know what to do in an emergency situation [ 4 ]  Because I thought it was reasonable in general [ 5 ]  Because it was an offer in school [ 6 ]  *(do not read out loud)* other reasons [ 6 ]  *(do not read out loud)* I don’t know [ 98 ] | 🡺D6  🡺D6  🡺D6  🡺D6  🡺D6  🡺D6  🡺D6  🡺D6 |
| D5  D5a  D5b  D5c  D5d  D5e  D5f  D5z | Why didn’t you ever participate in a first-aid-training?  *(Int.: multiple responses possible)*  Missing Information about first-aid-training [ 1 ]  Courses were too expensive [ 2 ]  I did not have time [ 3 ]  I did not have a motivation to participate [ 4 ]  I know enough about first-aid [ 5 ]  I am to old for something like that [ 6 ]  *(do not read out loud)* other reasons [ 6 ]  *(do not read out loud)* I don’t know [ 98 ] |  |
| D6 | There are some very short courses that just inform about resuscitation after cardiac arrest.  Have you ever participated in such a course?  Yes [ 1 ]  No [ 2 ]  *(do not read out loud)* Multiple times [ 3 ]  *(do not read out loud)* I don’t know [ 98 ] |  |
| D7  D8  D9 | Next I want to know how probable it is that you will participate in a first-aid-training within the next 12 month?  I will read to you three possibilities and you would like you to tell me please, how you estimate the probability that you will participate in a first-aid-training on a scale from 1 to 6. The value 1 would mean „very likely“ and 6 „unlikely“.   \|  \| very  likely \| \| \| unlikely \| \| \| spontaneously: I wouldn’t  participate in a training \| I don’t  know \| \| --- \| --- \| --- \| --- \| --- \| --- \| --- \| --- \| --- \| \| A first-aid-training with 16 hours of training \| [ 1 ] \| [ 2 ] \| [ 3 ] \| [ 4 ] \| [ 5 ] \| [ 6 ] \| [ 7 ] \| [ 98 ] \| \| A first-aid-refresher course with 8 hours of training \| [ 1 ] \| [ 2 ] \| [ 3 ] \| [ 4 ] \| [ 5 ] \| [ 6 ] \| [ 7 ] \| [ 98 ] \| \| A resuscitation-training with 2 hours of training \| [ 1 ] \| [ 2 ] \| [ 3 ] \| [ 4 ] \| [ 5 ] \| [ 6 ] \| [ 7 ] \| [ 98 ] \| | 7 = D10 |
| D10  D10a  D10b  D10c  D10d  D10e  D10f  D10g  D10z | Beside first-aid-trainings, there are further sources for information about resuscitation in case of cardiac arrest. I will read out some possibilities. Please tell me, whether you used any of these within the last 12 month.  *(multiple responses possible)*  Booklets and flyers......... [ 1 ]  Articles in newspapers or magazines [ 2 ]  Radio broadcasts [ 3 ]  Reports on TV [ 4 ]  Websites [ 5 ]  Posters [ 6 ]  Conversation with family and friends [ 7 ]  *(do not read out loud)* other sources [ 8 ]  *(do not read out loud)* I don’t know [ 98 ] | 🡺 F1  🡺 F1  🡺 F1  🡺 F1  🡺 D11  🡺 F1  🡺 F1  🡺 F1  🡺 F1 |
| D11  D11a  D11b  D11c  D11c | Did you use the following websites?  *(multiple response possible)*  www.einlebenretten.de [ 1 ]  www.100-pro-reanimation.de [ 2 ]  other website [ 3 ]  *(do not read out loud)* I don’t know [ 98 ] |  |
| **F** | **Demography** |  |
| F1 | Finally I want to ask for some information about you:  How old are you? ______ years  *(do not read out loud)* no answer [ 99 ] |  |
| F2 | What is your family status?  *(do not read out loud)* married/ civil union [ 1 ]  Single [ 2 ]  Divorced [ 3 ]  Widowed [ 4 ]  Other status [ 5 ]  No answer [ 99 ] |  |
| F3 | How many people live in your household - yourself included?  *(do not read out loud)* One person [ 1 ]  Two persons [ 2 ]  Three persons [ 3 ]  Four persons [ 4 ]  Five persons [ 5 ]  Six persons [ 6 ]  More than six persons [ 7 ]  I don’t know [ 98 ]  No answer [ 99 ] | 🡺 D10 |
| F4  F4a  F4b  F4c  F4d  F4e  F4z | What kind of people do you live with?  **(Int.: multiple responses possible)**  Spouse/partner [ 1 ]  Child/ Children [ 2 ]  Further family members (parents, siblings, etc.) [ 3 ]  Roommates [ 4 ]  Other people [ 5 ]  *(do not read out loud)* no answer [ 99 ] | Wenn F4_2≠1  🡺 F5 |
| F5 | How many children are younger than 14 years?    _______ Child/Children  *(do not read out loud)* no answer [ 99 ] |  |
| F6 | What is your highest educational degree?  *(do not read out loud)* No qualifications [ 1 ]  Elementary school / lower secondary school [ 2 ]  Intermediate school leaving certificate [ 3 ]  Vocational college diploma [ 4 ]  University entry qualification [ 5 ]  Other educational certificate [ 6 ]  Still student [ 7 ]  No answer [ 99 ] | 🡺F8 |
| F7 | Which occupational degree do you have?  University degree [ 1 ]  Polytechnical degree [ 2 ]  Foreman, technician or comparable degree [ 3 ]  Industrial or agricultural traineeship [ 4 ]  Commercial traineeship [ 5 ]  (still) no degree [ 6 ]  *(do not read out loud)* internship, voluntary service [ 7 ]  Skilled worker degree [ 8 ]  Occupational-operating training period with diploma [ 9 ]  Different degree [ 10 ]  I don’t know [ 98 ]  No answer [ 99 ] |  |
| F8  F8a  F8b  F8c  F8d  F8e  F8z | Are you or have you ever been full-time working or volunteering in medical fields or emergency services?  *(multiple responses possible, do not read out loud)*  Full-time in medical field [ 1 ]  Voluntary work in medical field [ 2 ]  Full-time in emergency services [ 3 ]  Voluntary work in emergency services [ 4 ]  Not working in medical or ambulance services [ 5 ]    I don’t know [ 98 ] |  |
| F9 | What is the monthly net income of your household? What I mean is the amount of money after subtracting taxes and social charges.  *(approximate rating; in flat-share: just own income)*  Less than 1000 Euro [ 1 ]  1000 to 2000 Euro [ 2 ]  2001 to 3000 Euro [ 3 ]  3001 to 4000 Euro [ 4 ]  4001 to 5000 Euro [ 5 ]  More than 5000 Euro [ 6 ]  *(do not read out loud)* I don’t know [ 98 ]  No answer [ 99 ] |  |
|  | **END**  **Do you have any questions about this survey? Thank you for this conversation.** |  |
| F10 | Gender of the informant  Female [ 1 ]  Male [ 2 ] |  |
